# Blood cell counts can predict adverse events of immune checkpoint inhibitors: A systematic review and meta-analysis

**DOI:** 10.3389/fimmu.2023.1117447

**Published:** 2023-03-07

**Authors:** Juyue Zhou, Zhonghai Du, Jie Fu, Xiuxiu Yi

**Affiliations:** ^1^ Graduate Institute, Shandong University of Traditional Chinese Medicine, Jinan, Shandong, China; ^2^ Department of Oncology, Weifang Hospital of Traditional Chinese Medicine, Weifang, Shandong, China

**Keywords:** immune checkpoint inhibitor, immunotherapy, adverse effect, blood cell count, risk factor

## Abstract

**Background:**

Cancer is concerning owing to its high mortality rate. Consequently, methods of prolonging the life of patients with cancer have become the primary focus of attention research. In recent years, immune checkpoint inhibitors (ICIs) have achieved good clinical efficacy as antitumor drugs; however, their severe adverse effects have made their use challenging. In order to clarify the predictors of adverse effects, scientists have conducted a series of studies. Blood counts can potentially monitor risk factors associated with the occurrence of immune-related adverse events (irAEs). Herein, a meta-analysis was performed to clarify further the guiding significance of blood counts in the clinical setting.

**Methods:**

Studies that satisfied the inclusion criteria were obtained by searching the database. Included studies were those in which irAEs had been observed, and evidence of an association between blood counts and irAEs was reported. The included ones were evaluated for quality. In addition to sensitivity analysis and subgroup analysis, a meta-analysis was performed using the odds ratio (OR) and 95% confidence interval (CI) for each study.

**Results:**

A total of 18 articles were included in our study. The analyses were performed separately according to different blood cell count indicators. The blood cell count metrics associated with irAEs were: absolute eosinophil count, neutrophil: lymphocyte ratio, and platelet: lymphocyte ratio.

**Conclusion:**

Our review and meta-analysis of studies suggest that absolute eosinophil count, neutrophil: lymphocyte ratio, and platelet: lymphocyte ratio may serve as predictors of the emergence of irAEs. Given the small number of studies focusing on the relationship between patient blood cell counts and the risk of irAEs, future studies need to further explore the mechanisms of occurrence and potential associations.

## 1 Introduction

Cancer treatments deserve more attention due to the rising incidence and high mortality rate. Unlike conventional chemotherapeutic drugs that kill cells directly, immune checkpoint inhibitors, including cytotoxic T-lymphocyte antigen-4 (CTLA-4), programmed death-1 (PD-1), and programmed death-ligand 1 (PD-L1) inhibitors, follow a different therapeutic pathway (1). The first approved CTLA-4 inhibitors enhance T-cell activation and induce sustained antitumor responses by blocking the CTLA-4 checkpoint that inhibits T-cell responses (2–5). Anti-PD-1/PD-L1 inhibitors activate T-cell toxicity and eliminate tumor cells by blocking checkpoint-induced tumor escape (4, 5). These ICIs have shown their powerful therapeutic capabilities in the treatment of tumors, such as those in breast cancer, gastric cancer, angiosarcoma, and prostate cancer (1, 4). However, the antitumor effects of ICIs are accompanied by damage to normal cells, and which may upset the homeostasis of the human immune system, which maintains a unique steady state of normal conditions (3, 6). Immune adverse reactions in the skin, liver, gastrointestinal tract, lungs, and endocrine organs are common (7). Due to the possibility of drug discontinuation and even death as a result of adverse reactions (8), many researchers analyze the potential predictors of the occurrence of adverse reactions in order to prevent and control them early. These several relevant factors have been shown to have a possible association with adverse reactions, such as drug selection, gender, age, laboratory tests, pre-existing autoimmune disease (pAID), PD-L1 expression, and tumor mutation burden (TMB) (9–14). However, studying these factors is difficult to use widely implement in clinical practice due to low accuracy or the involvement of expensive tests. Therefore, blood cell count levels that can be measured repeatedly and inexpensively are gaining importance, and we focused on determining the relationship between them.

## 2 Materials and methods

### 2.1 Literature search

We conducted a comprehensive search of PubMed, Embase, and Web of Science databases from when the database was created until December 1, 2022. The search keywords include “immune checkpoint inhibitors”, “immune checkpoint inhibitor-related adverse reaction”, “risk factor”, “blood biomarker”, “eosinophil”, “neutrophil”, “lymphocyte”, “monocyte”, etc. The specific search formula can be found in the appendix.

### 2.2 Inclusion and exclusion criteria

The inclusion criteria were as follows (1): Patients had malignant tumors (2); The study was a randomized clinical trial, retrospective clinical study, or case-control study; (3) Patients received ICI therapy; (4) The association between blood cell counts and the occurrence of irAEs was assessed by the authors; and (5) Definite odds ratio (OR) and 95% CI. If no OR value is specified, at least specific values that can be calculated should be provided.

The exclusion criteria were as follows: (1) The study type was animal experiments; (2) Risk factors for the occurrence of irAEs were not assessed; (3) Blood counts were not considered to be risk factors; and (4) Patients had hematologic tumors.

### 2.3 Data extraction

After an initial screening based on the title and abstract of the publication and excluding any irrelevant literature, the remaining literature was read in total, and those that should be included were selected.

Two independent investigators identified the included papers, and a third investigator resolved disagreements, if any. The following information was extracted: title, author, publication year, country, patient number, patient origin, research type, risk factors, type of irAEs, optimal cutoff value, OR, and 95% CI. If univariate and multivariate regression analyses were conducted, ORs were calculated using the multivariate regression analysis data. When OR values were unavailable, crude OR values and 95% CIs were calculated based on frequency tables.

### 2.4 Quality assessment

The quality in prognosis studies (QUIPS) tool was used to evaluate the risk of bias. Briefly, two independent researchers divided the literature into low, moderate, and high risk-of-bias categories. Any disagreement was discussed by adding a third person until an agreement was reached.

### 2.5 Data analysis

The same risk factor was analyzed using the “metan” command in Stata17. Data included crude or adjusted ORs and 95% CIs, with ORs adjusted for confounders selected whenever possible. Additionally, the heterogeneity test was conducted, and I^2^ > 50% was considered highly heterogeneous.

## 3 Results

### 3.1 Research selection

**Figure 1:**
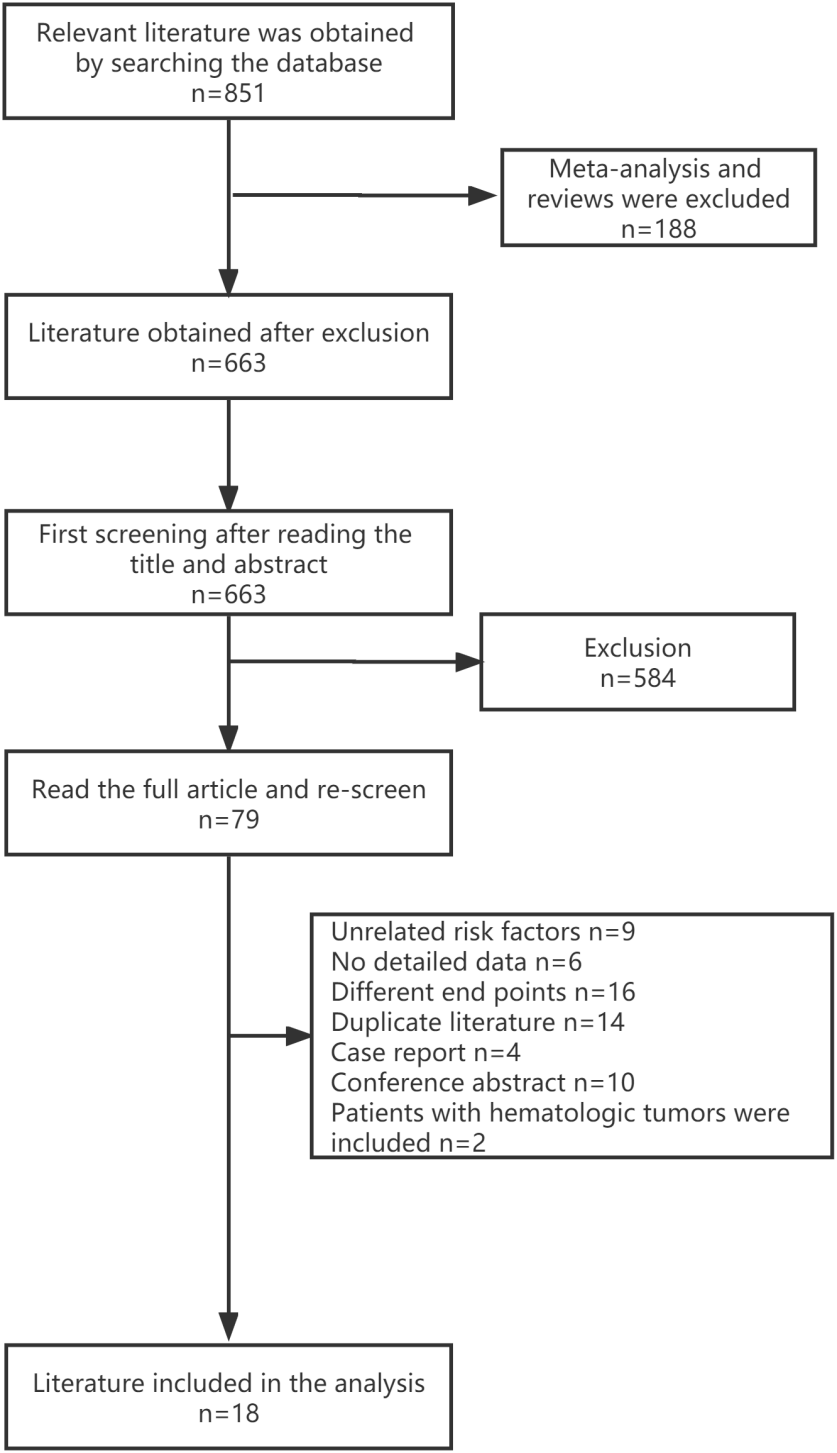
Flowchart of study screening.

The three databases were searched with the set search formula, and 851 publications fit the criteria. Among them, 188 meta-analyses and systematic reviews were excluded. After reading the title and abstract, 584 publications were excluded. Further filtering was done by reading the full text. Finally, 18 studies were included. Details can be found in the flow chart. (**Figure 1**)

### 3.2 Research characteristics

A total of 18 studies (11, 15–31) were included in the analysis. These studies were published between 2019 to 2022. Nine studies took place in China, six in Japan, one in Singapore, one in Italy, one in Canada, which shows that most of these studies were involved the Asian population. A total of 3579 patients were included in the study. Previous treatment was described in four articles (15–18), one of which included previous antitumor regimens as one of the criteria when including patients (16). Six articles included only patients who received ICI monotherapy (15, 16, 19–22). The remaining studies were unrestricted, and they included patients who received ICI in combination with other antitumor treatments. With immune checkpoint inhibitor administration as the node, blood cell counts from all studies were evaluated. Data from either the beginning or end of immunotherapy were chosen for analysis. The types of blood counts reported are as follows: absolute eosinophil count (AEC), derived neutrophil: lymphocyte ratio (dNLR), neutrophil: lymphocyte ratio (NLR), absolute neutrophil count (ANC), absolute lymphocyte count (ALC), absolute monocyte count (AMC), platelet count (PLT), monocyte: lymphocyte ratio (MLR), platelet: lymphocyte ratio (PLR), etc. Eight studies showed substantial truncation values (11, 15–17, 20, 23, 25, 28). Eleven studies reported the use of receiver operating characteristic (ROC) curve analysis to calculate the optimal cutoff value (11, 15–17, 19–21, 23–26). In all included reports, skin adverse reactions were the most common (23.34%), followed by endocrine adverse reactions (22.81%), immune pneumonia (13.50%), hepatic adverse reactions (10.25%), and gastrointestinal adverse reactions (9.23%). All studies were retrospective. More details are shown in **Supplementary Table 1**.

### 3.3 Bias risk assessment

QUIPS tool was used to assess the risk of bias; detailed entries include study participation, study attrition, prognostic factor measurement, outcome measurement, study confounding, statistical analysis and reporting. After assessing the articles, seven were classified as high risk of bias, six as medium risk, and five as low risk. (**Supplementary Figure 1**)

### 3.4 Meta-analysis of major risk factors

The same risk factor reported in at least two publications was included in the study, with preference given to data reporting cutoff values. Further, the results of multifactorial analyses were prioritized for inclusion, and if unavailable, single-factor results were considered for inclusion. Available for analysis are AEC, NLR, and PLR.

### 3.5 AEC

**Figure 2:**
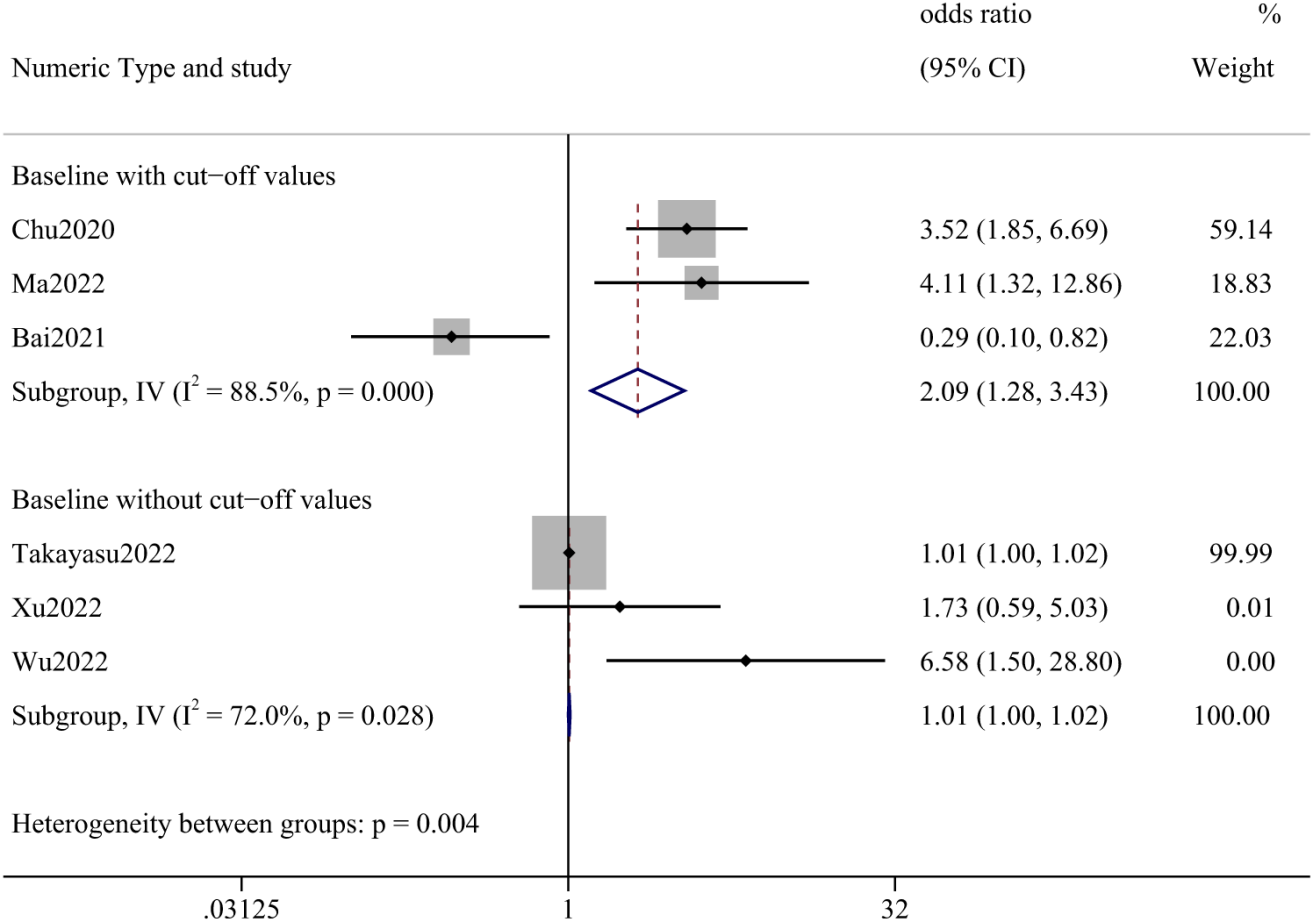
Forest plot of correlation between AEC and incidence of irAEs.

Six papers described AEC, three of which indicated a cut-off value. We analyzed articles that provided a cut-off value separately and those that did not. The meta-analysis, including only cut-off values, yielded an OR of 2.09 (95% CI: 1.28–3.43). Studies that did not report cut-off values had an OR of 1.01 (95% CI: 1.00–1.02). (**Figure 2**) In this analysis, 1202 cases were involved, the most frequent being lung cancer (70.97%), followed by renal and urological tumors (10.73%), malignant melanoma (4.99%), and head and neck tumors (4.41%).

### 3.6 NLR

**Figure 3:**
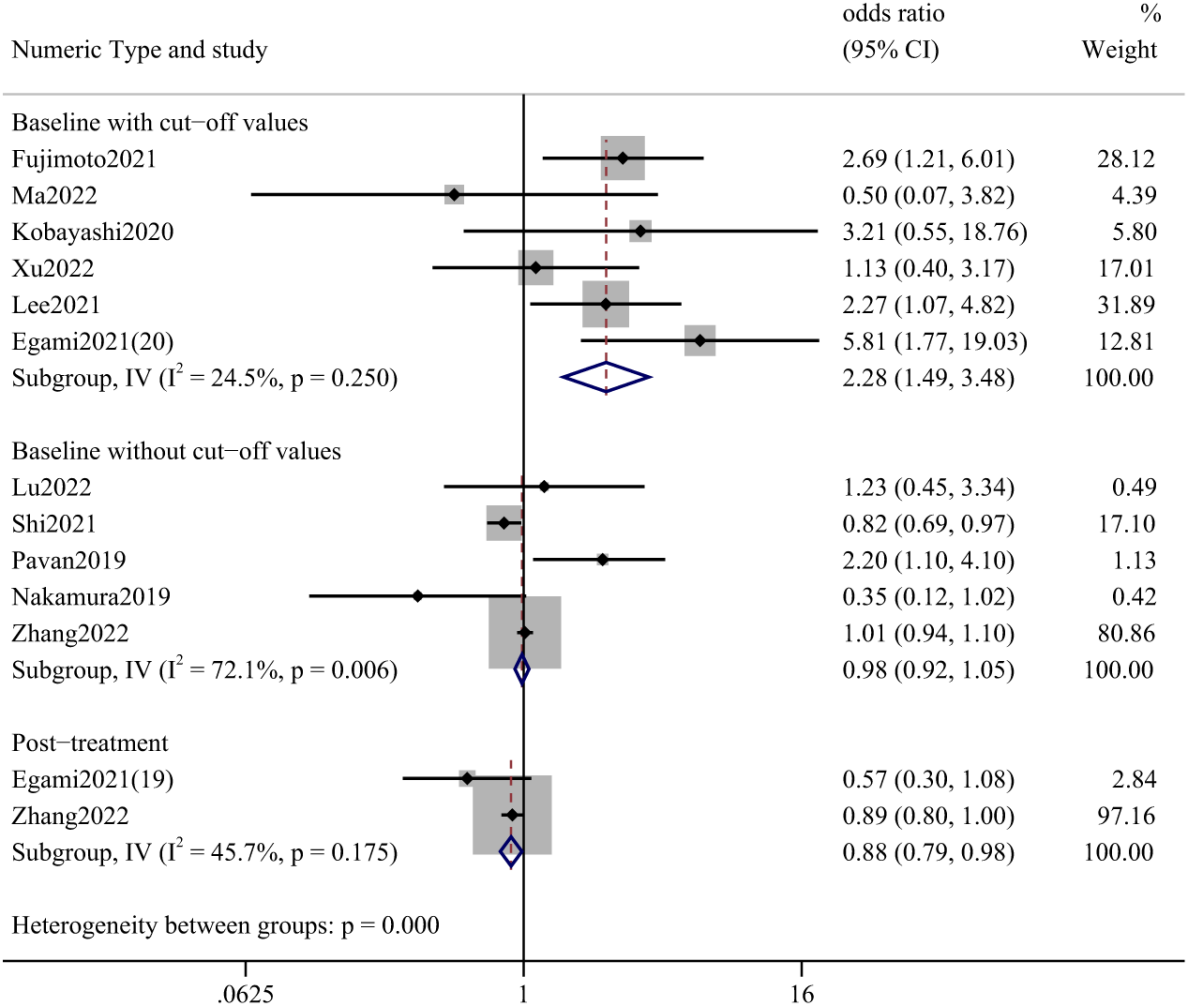
Forest plot of correlation between NLR and incidence of irAEs.

The effect of pre-treatment NLR on the incidence of irAEs was mainly described in studies reporting NLR; two articles reported the effect of post-treatment values. We divided the analysis into three parts: baseline with and without cutoff values and post-treatment data. The most statistically significant studies were those accompanied by a truncated value at baseline, with an OR of 2.28 (95%CI: 1.49–3.48). (**Figure 3**) The studies that provided cut-off values included a total of 1116 cases, with lung cancer (87.81%), renal and urinary tumors (5.29%), head and neck tumors (1.52%), and esophageal cancer (1.52%).

### 3.7 PLR

**Figure 4:**
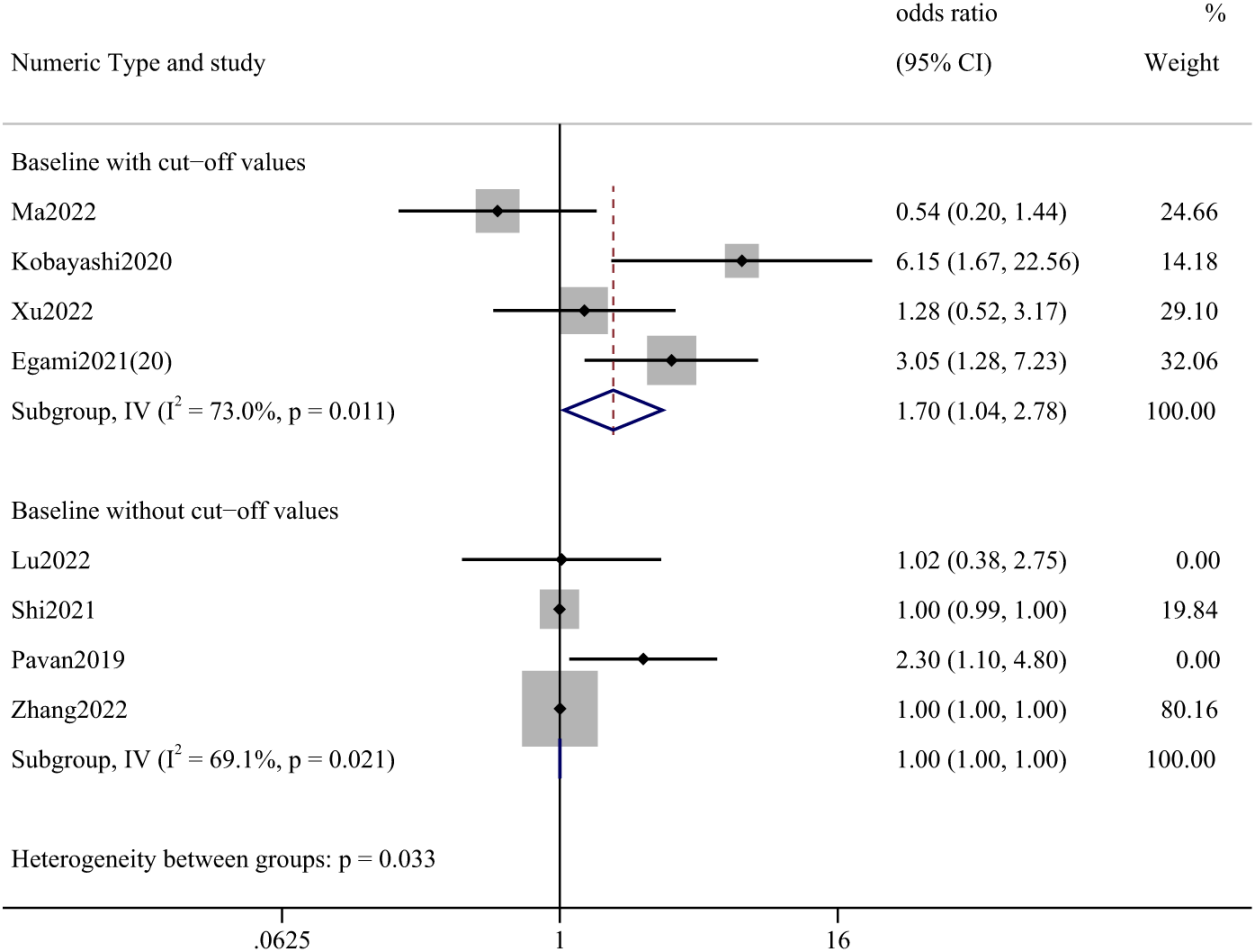
Forest plot of correlation between PLR and incidence of irAEs.

Among the articles presenting PLR, one presenting post-treatment values was not analyzed. The study that reported a cutoff value was statistically significant with an OR of 1.70 (95% CI: 1.04-2.78). (**Figure 4**) A total of 907 cases were involved in the studies that reported cut-off values, including lung cancer (86.44%), renal and urological tumors (6.28%), esophageal cancer (1.87%), and liver cancer (1.21%).

### 3.8 Analysis of cut-off values

We present data from studies that reported cut-off values and analyzed them further. (**Table 1**) Studies involving AEC have concluded that higher than optimal threshold values are associated with the occurrence of adverse reactions in patients. Of the studies that reported NLR cutoff values, only one concluded that being above the cutoff value caused an increased incidence of adverse reactions (20), whereas the remaining studies concluded that being below the cutoff value was associated with an event rate of a combined OR 2.52 (95%CI:1.49-4.52). While in PLR-related studies, PLR above the cutoff (OR: 1.43, 95% CI: 0.75–2.75) was analyzed, but considering that one of the articles did not show a correlation and the comparison of OR values, we think the below the cutoff has a higher correlation with event occurrence. Further analysis of the specific range of values chosen can be seen in the subgroup analysis.

**Table 1:** Table of cut-off values.

Study	Risk Factors	Cut-off value	OR	LCI	UCI	P value
Chu2020	AEC	≥0.125	3.518	1.851	6.686	<0.001
Ma2022	AEC	> 0.045	4.114	1.32	12.858	0.014
Bai2021	AEC	< 0.175	0.29	0.1	0.82	0.020
Fujimoto2021	NLR	<2.86	2.69	1.21	6.01	0.016
Ma2022	NLR	>8.58	0.501	0.066	3.816	0.505
Kobayashi2020	NLR	< 3.4	3.21	0.55	18.76	0.194
Egami2021 (20)	NLR	> 2.3	5.81	1.77	19.03	0.004
Lee2021	NLR	<3	2.27	1.07	4.82	0.034
Ma2022	PLR	≥180.68	0.537	0.2	1.44	0.216
Kobayashi2020	PLR	< 156	6.15	1.67	22.56	0.006
Egami2021 (20)	PLR	> 165	3.05	1.28	7.23	0.011

### 3.9 Other risk factors

Fewer articles presented truncation values of ANC, ALC, AMC, PLT, and LMR. Therefore, we did not perform further analysis.

### 3.10 Risk of bias

Heterogeneity was observed in the meta-analysis of all three indicators. Funnel plots indicate possible publication bias, and studies with positive results are more likely to be selected. (Figure not shown)

### 3.11 Sensitivity analysis

Sensitivity analyses were performed by sequentially eliminating individual studies to clarify whether each study affected the overall results. The analysis gave stable results for sensitivity analysis in the NLR and PLR studies that reported cutoff values and in the AEC studies that did not report cutoff values. In contrast, sensitivity analysis showed that the results might be unstable and not statistically significant in other studies. (Figure not shown)

### 3.12 Subgroup analysis

Subgroup analyses were performed for PLR and NLR based on country, the number of cases, and cutoff values. Due to the limited number of studies, the remaining risk factors were not included in these additional analyses. The country-based subgroups showed that NLR was more significant in the incidence of irAEs in Japanese patients with an OR of 3.40 (95% CI: 1.82-6.32), significantly higher than that in the Chinese population had an OR of 0.96 (95%CI: 0.38-2.40). The number of studies that did not report NLR cutoff values was insufficient for subgroup analysis. PLR contributed more significantly to the incidence of irAEs in the Japanese population than in the Chinese population, with an OR of 3.78 (95% CI:1.84-7.78) vs 0.86 (95% CI:0.44-1.67), respectively. We further performed a stratified analysis of the number of patients included in the study. Statistical significance was most significant when the number of people included in studies on NLR was less than 100. Effect values did not differ significantly between patient numbers in the PLR group where cutoff values were reported. In addition, four articles reported NLR cutoff values between 2.5 and 3.5, and their OR was calculated to be 2.13 (95%CI: 1.34-3.40). Due to the limited number of studies, there may be inaccuracies in the analysis results for each subgroup. All the above results are represented in **Supplementary Table 2**.

## 4 Discussion

This meta-analysis aimed to investigate whether blood cell counts can predict the occurrence of adverse reactions triggered during cancer treatment with ICIs and to determine the best predictors. A study of the literature that met the inclusion criteria showed that AEC, NLR, and PLR could be predictors of adverse reactions.

In addition to the fact that blood counts appear to be a predictor of irAEs, the underlying mechanisms are worth mentioning.

To interrupt this process of immune evasion caused by cancer cell-induced suppression of the immune system by immunosuppressive cells and immune checkpoints (32), researchers focused on immune checkpoints. ICIs block the signaling of suppressed T cells and restore the anti-tumor effects of the cells while blocking negative regulation of T cells and promoting higher levels of pro-inflammatory factors and activation of anti-tumor immune responses by T cells (32–35). After immune checkpoint blockade, the balance between autoimmunity and immune tolerance is lost, self-tolerance is disrupted, and adverse reactions occur (36). Possible mechanisms include enhanced activation and proliferation of effector CD8+ T cells by CTLA and PD-1/PD-L1 inhibitors, elimination of regulatory T (Treg) cell function, induction of inflammatory cytokines, possible enhancement of humoral autoimmunity, and induction of normal tissue damage (37, 38).

The indicators mentioned in our study are closely linked to various aspects of the mechanisms of irAEs occurrence. Eosinophils have an immunomodulatory role in defining the T-cell pool and participating in apoptosis (39) and have anti-tumor effects themselves. ICIs increase the infiltration of eosinophils into tumors, acting through direct mechanisms, such as releasing cytotoxic proteins and expressing natural killer cell activation receptors to induce cytotoxicity, and indirect mechanisms, such as releasing interferon gamma to promote anti-tumor immunity (40). However, while proven to be a good prognostic factor in solid tumors, adverse reactions were found to be more common in patients with high AEC at baseline (41). This finding was confirmed in our meta-analysis, where patients above the AEC cut-off had a greater chance of adverse reactions.

PLR and NLR are recognized inflammatory markers for the early identification of cancer and to assist in the assessment of efficacy and prognosis after checkpoint inhibitor therapy (42–44). Low PLR and NLR were more predictive of adverse events in our study. Platelets, as primary inflammatory effector cells, are involved in innate and adaptive immune responses (45), and stimulation of Toll-like receptor (TLR)-containing platelets by pathogens causes activation of neutrophils and promotes recruitment and interaction. Activated platelets are able to secrete pro-inflammatory factors involved in pathogen killing (46). Neutrophils, which are also involved in the immune process, affect the cytotoxic effect of lymphocytes, thus weakening the antitumor effect due to lymphocyte activation during ICI treatment (47). Thus, low neutrophil values favor the antitumor effects of drug-activated lymphocytes and increase the incidence of irAEs (15). Lymphocyte counts have been shown to be associated with multiple adverse effects (48), and damage to their own tissues following their activation is a possible mechanism for the development of adverse effects (15), with clonal expansion of CD8+ T lymphocytes seen before symptoms are present (49). Low PLR and NLR represent low platelet levels, low neutrophil levels, and high lymphocyte levels, reflecting the maintenance of antitumor effects and predicting an increased risk for developing irAEs.

This review has several limitations. Publication bias is prevalent and may be related to factors such as the small number of included studies and the predominance of positive results in the reported studies. Nevertheless, sensitivity analyses suggest that associations observed in the PLR and NLR groups are robust. In contrast, analyses in the AEC group have suggested that the association between the two may have been overestimated. Heterogeneity, mainly methodological heterogeneity, should be considered. Specific assays were not included in any of the clinical designs in the studies, and the effect due to differences in testing instruments or reagents may have been underestimated. In addition, most studies did not exclude other factors contributing to elevated inflammatory marker levels; these should be considered in the analysis. Due to the small number of included studies, some limitations are seen in the data from the subgroup analysis. There needed to be more studies on some indicators; therefore, adequate study data could not be obtained. In addition, most of the studies included were from the Asian region, and possible ethnic differences were not examined.

Therefore, the relationship between blood cell counts and immunotoxicity needs further confirmed and investigated.

## 5 Conclusion

Our review and meta-analysis suggest that absolute eosinophil count, neutrophil-to-lymphocyte ratio, and platelet-to-lymphocyte ratio are associated with a high risk of irAEs. This relationship may help assess the therapeutic risk in patients receiving immune checkpoint inhibitors, and patients at higher therapeutic risk should be closely monitored. Early and adequate treatment of irAEs may improve the prognosis of patients who have developed immunotoxicity. Given the lack of studies focusing on the relationship between blood tests and the risk of irAEs, future studies need to further explore the mechanisms of occurrence and underlying associations.

## Data availability statement

The original contributions presented in the study are included in the article/**Supplementary Material**. Further inquiries can be directed to the corresponding author.

## Author contributions

ZD contributed to the study concept. JZ refined the study design. JZ and JF searched and screened the included literature. All authors contributed to the article and approved the submitted version.

## References

[1] von Itzstein MS Khan S Gerber DE . Investigational biomarkers for checkpoint inhibitor immune-related adverse event prediction and diagnosis. Clin Chem (2020) 66(6):779–93. doi: 10.1093/clinchem/hvaa081 PMC7259479 32363387

[2] Zhang H Dai Z Wu W Wang Z Zhang N Zhang L . Regulatory mechanisms of immune checkpoints pd-L1 and ctla-4 in cancer. J Exp Clin Cancer Res (2021) 40(1):184. doi: 10.1186/s13046-021-01987-7 34088360 PMC8178863

[3] Morad G Helmink BA Sharma P Wargo JA . Hallmarks of response, resistance, and toxicity to immune checkpoint blockade. Cell (2021) 184(21):5309–37. doi: 10.1016/j.cell.2021.09.020 PMC8767569 34624224

[4] Tang Q Chen Y Li X Long S Shi Y Yu Y . The role of pd-1/Pd-L1 and application of immune-checkpoint inhibitors in human cancers. Front Immunol (2022) 13:964442. doi: 10.3389/fimmu.2022.964442 36177034 PMC9513184

[5] Zhang Y Zhang Z . The history and advances in cancer immunotherapy: Understanding the characteristics of tumor-infiltrating immune cells and their therapeutic implications. Cell Mol Immunol (2020) 17(8):807–21. doi: 10.1038/s41423-020-0488-6 PMC7395159 32612154

[6] Yang H Yao Z Zhou X Zhang W Zhang X Zhang F . Immune-related adverse events of checkpoint inhibitors: Insights into immunological dysregulation. Clin Immunol (2020) 213:108377. doi: 10.1016/j.clim.2020.108377 32135278

[7] Tang SQ Tang LL Mao YP Li WF Chen L Zhang Y . The pattern of time to onset and resolution of immune-related adverse events caused by immune checkpoint inhibitors in cancer: A pooled analysis of 23 clinical trials and 8,436 patients. Cancer Res Treat (2021) 53(2):339–54. doi: 10.4143/crt.2020.790 PMC8053869 33171025

[8] Khan OF Monzon J . Diagnosis, monitoring, and management of adverse events from immune checkpoint inhibitor therapy. Curr Oncol (2020) 27(Suppl 2):S43–50. doi: 10.3747/co.27.5111 PMC7194002 32368173

[9] Poto R Troiani T Criscuolo G Marone G Ciardiello F Tocchetti CG . Holistic approach to immune checkpoint inhibitor-related adverse events. Front Immunol (2022) 13:804597. doi: 10.3389/fimmu.2022.804597 35432346 PMC9005797

[10] Jing Y Zhang Y Wang J Li K Chen X Heng J . Association between sex and immune-related adverse events during immune checkpoint inhibitor therapy. J Natl Cancer Inst (2021) 113(10):1396–404. doi: 10.1093/jnci/djab035 33705549

[11] Chu X Zhao J Zhou J Zhou F Jiang T Jiang S . Association of baseline peripheral-blood eosinophil count with immune checkpoint inhibitor-related pneumonitis and clinical outcomes in patients with non-small cell lung cancer receiving immune checkpoint inhibitors. Lung Cancer (2020) 150:76–82. doi: 10.1016/j.lungcan.2020.08.015 33080551

[12] Xu Y Fu Y Zhu B Wang J Zhang B . Predictive biomarkers of immune checkpoint inhibitors-related toxicities. Front Immunol (2020) 11:2023. doi: 10.3389/fimmu.2020.02023 33123120 PMC7572846

[13] Asada M Mikami T Niimura T Zamami Y Uesawa Y Chuma M . The risk factors associated with immune checkpoint inhibitor-related pneumonitis. Oncology (2021) 99(4):256–9. doi: 10.1159/000512633 33477139

[14] Placais L Dalle S Dereure O Trabelsi S Dalac S Legoupil D . Risk of iraes in patients with autoimmune diseases treated by immune checkpoint inhibitors for stage III or iv melanoma: Results from a matched case-control study. Ann Rheum Dis (2022) 81(10):1445–52. doi: 10.1136/ard-2022-222186 35788496

[15] Fujimoto A Toyokawa G Koutake Y Kimura S Kawamata Y Fukuishi K . Association between pretreatment neutrophil-to-Lymphocyte ratio and immune-related adverse events due to immune checkpoint inhibitors in patients with non-small cell lung cancer. Thorac Cancer (2021) 12(15):2198–204. doi: 10.1111/1759-7714.14063 PMC8327687 34173724

[16] Kobayashi K Iikura Y Hiraide M Yokokawa T Aoyama T Shikibu S . Association between immune-related adverse events and clinical outcome following nivolumab treatment in patients with metastatic renal cell carcinoma. In Vivo (2020) 34(5):2647–52. doi: 10.21873/invivo.12083 PMC7652517 32871795

[17] Bai R Chen N Chen X Li L Song W Li W . Analysis of characteristics and predictive factors of immune checkpoint inhibitor-related adverse events. Cancer Biol Med (2021) 18(4):1118–33. doi: 10.20892/j.issn.2095-3941.2021.0052 PMC8610160 34259422

[18] Pavan A Calvetti L Dal Maso A Attili I Del Bianco P Pasello G . Peripheral blood markers identify risk of immune-related toxicity in advanced non-small cell lung cancer treated with immune-checkpoint inhibitors. Oncologist (2019) 24(8):1128–36. doi: 10.1634/theoncologist.2018-0563 PMC6693718 31015312

[19] Egami S Kawazoe H Hashimoto H Uozumi R Arami T Sakiyama N . Absolute lymphocyte count predicts immune-related adverse events in patients with non-Small-Cell lung cancer treated with nivolumab monotherapy: A multicenter retrospective study. Front Oncol (2021) 11:618570. doi: 10.3389/fonc.2021.618570 34123782 PMC8190379

[20] Egami S Kawazoe H Hashimoto H Uozumi R Arami T Sakiyama N . Peripheral blood biomarkers predict immune-related adverse events in non-small cell lung cancer patients treated with pembrolizumab: A multicenter retrospective study. J Cancer (2021) 12(7):2105–12. doi: 10.7150/jca.53242 PMC7974524 33754009

[21] Nakamura Y Tanaka R Maruyama H Ishitsuka Y Okiyama N Watanabe R . Correlation between blood cell count and outcome of melanoma patients treated with anti-Pd-1 antibodies. Jpn J Clin Oncol (2019) 49(5):431–7. doi: 10.1093/jjco/hyy201 30753621

[22] Zhang Z Xie T Qi C Zhang X Shen L Peng Z . Peripheral blood biomarkers predictive of efficacy outcome and immune-related adverse events in advanced gastrointestinal cancers treated with checkpoint inhibitors. Cancers(Basel) (2022) 14(15):3736. doi: 10.3390/cancers14153736 35954401 PMC9367581

[23] Ma Y Ma X Wang J Wu S Wang J Cao B . Absolute eosinophil count may be an optimal peripheral blood marker to identify the risk of immune-related adverse events in advanced malignant tumors treated with pd-1/Pd-L1 inhibitors: A retrospective analysis. World J Surg Oncol (2022) 20(1):242. doi: 10.1186/s12957-022-02695-y 35897018 PMC9331074

[24] Takayasu S Mizushiri S Watanuki Y Yamagata S Usutani M Nakada Y . Eosinophil counts can be a predictive marker of immune checkpoint inhibitor-induced secondary adrenal insufficiency: A retrospective cohort study. Sci Rep (2022) 12(1):1294. doi: 10.1038/s41598-022-05400-x 35079086 PMC8789805

[25] Xu H Feng H Zhang W Wei F Zhou L Liu L . Prediction of immune-related adverse events in non-small cell lung cancer patients treated with immune checkpoint inhibitors based on clinical and hematological markers: Real-world evidence. Exp Cell Res (2022) 416(1):113157. doi: 10.1016/j.yexcr.2022.113157 35427598

[26] Lu X Wan J Shi H . Platelet-to-lymphocyte and neutrophil-to-lymphocyte ratios are associated with the efficacy of immunotherapy in stage III/IV non-small cell lung cancer. Oncol Lett (2022) 24(2):266. doi: 10.3892/ol.2022.13386 35782904 PMC9247654

[27] Shi Y Liu X Liu J Zhang D Liu X Yue Y . Correlations between peripheral blood biomarkers and clinical outcomes in advanced non-small cell lung cancer patients who received immunotherapy-based treatments. Transl Lung Cancer Res (2021) 10(12):4477–93. doi: 10.21037/tlcr-21-710 PMC8743518 35070755

[28] Lee PY Oen KQX Lim GRS Hartono JL Muthiah M Huang DQ . Neutrophil-to-Lymphocyte ratio predicts development of immune-related adverse events and outcomes from immune checkpoint blockade: A case-control study. Cancers(Basel) (2021) 13(6):1308. doi: 10.3390/cancers13061308 33804050 PMC8001500

[29] Zhao L Li Y Jiang N Song X Xu J Zhu X . Association of blood biochemical indexes and antibiotic exposure with severe immune-related adverse events in patients with advanced cancers receiving pd-1 inhibitors. J Immunother (2022) 45(4):210–6. doi: 10.1097/CJI.0000000000000415 PMC8986630 35250004

[30] Wu Y Li D Wu M Yang Y Shen M Chen K . Peripheral absolute eosinophil count identifies the risk of serious immune-related adverse events in non-small cell lung cancer. Front Oncol (2022) 12:1004663. doi: 10.3389/fonc.2022.1004663 36313675 PMC9608122

[31] Ksienski D Wai ES Alex D Croteau NS Freeman AT Chan A . Prognostic significance of the neutrophil-to-Lymphocyte ratio and platelet-to-Lymphocyte ratio for advanced non-small cell lung cancer patients with high pd-L1 tumor expression receiving pembrolizumab. Transl Lung Cancer Res (2021) 10(1):355–67. doi: 10.21037/tlcr-20-541 PMC7867765 33569318

[32] Wang DR Wu XL Sun YL . Therapeutic targets and biomarkers of tumor immunotherapy: Response versus non-response. Signal Transduct Target Ther (2022) 7(1):331. doi: 10.1038/s41392-022-01136-2 36123348 PMC9485144

[33] Sweis RF Luke JJ . Mechanistic and pharmacologic insights on immune checkpoint inhibitors. Pharmacol Res (2017) 120:1–9. doi: 10.1016/j.phrs.2017.03.012 28323141 PMC5419683

[34] Buchbinder EI Desai A . Ctla-4 and pd-1 pathways: Similarities, differences, and implications of their inhibition. Am J Clin Oncol (2016) 39(1):98–106. doi: 10.1097/COC.0000000000000239 26558876 PMC4892769

[35] Goleva E Lyubchenko T Kraehenbuehl L Lacouture ME Leung DYM Kern JA . Our current understanding of checkpoint inhibitor therapy in cancer immunotherapy. Ann Allergy Asthma Immunol (2021) 126(6):630–8. doi: 10.1016/j.anai.2021.03.003 PMC8713301 33716146

[36] Young A Quandt Z Bluestone JA . The balancing act between cancer immunity and autoimmunity in response to immunotherapy. Cancer Immunol Res (2018) 6(12):1445–52. doi: 10.1158/2326-6066.CIR-18-0487 PMC6281171 30510057

[37] Tang L Wang J Lin N Zhou Y He W Liu J . Immune checkpoint inhibitor-associated colitis: From mechanism to management. Front Immunol (2021) 12:800879. doi: 10.3389/fimmu.2021.800879 34992611 PMC8724248

[38] Kim ST Chu Y Misoi M Suarez-Almazor ME Tayar JH Lu H . Distinct molecular and immune hallmarks of inflammatory arthritis induced by immune checkpoint inhibitors for cancer therapy. Nat Commun (2022) 13(1):1970. doi: 10.1038/s41467-022-29539-3 35413951 PMC9005525

[39] Weller PF Spencer LA . Functions of tissue-resident eosinophils. Nat Rev Immunol (2017) 17(12):746–60. doi: 10.1038/nri.2017.95 PMC5783317 28891557

[40] Grisaru-Tal S Itan M Klion AD Munitz A . A new dawn for eosinophils in the tumour microenvironment. Nat Rev Cancer (2020) 20(10):594–607. doi: 10.1038/s41568-020-0283-9 32678342

[41] Ramos-Casals M Flores-Chavez A Brito-Zeron P . Emerging role of eosinophils in immune-related adverse events related to therapy with immune checkpoint inhibitors. Pol Arch Intern Med (2021) 131(10):16092. doi: 10.20452/pamw.16092 34704705

[42] Liu J Li S Zhang S Liu Y Ma L Zhu J . Systemic immune-inflammation index, neutrophil-to-Lymphocyte ratio, platelet-to-Lymphocyte ratio can predict clinical outcomes in patients with metastatic non-Small-Cell lung cancer treated with nivolumab. J Clin Lab Anal (2019) 33(8):e22964. doi: 10.1002/jcla.22964 31282096 PMC6805305

[43] Graziano V Grassadonia A Iezzi L Vici P Pizzuti L Barba M . Combination of peripheral neutrophil-to-Lymphocyte ratio and platelet-to-Lymphocyte ratio is predictive of pathological complete response after neoadjuvant chemotherapy in breast cancer patients. Breast (2019) 44:33–8. doi: 10.1016/j.breast.2018.12.014 30611095

[44] Nøst TH Alcala K Urbarova I Byrne KS Guida F Sandanger TM . Systemic inflammation markers and cancer incidence in the uk biobank. Eur J Epidemiol (2021) 36(8):841–8. doi: 10.1007/s10654-021-00752-6 PMC8416852 34036468

[45] Dib PRB Quirino-Teixeira AC Merij LB Pinheiro MBM Rozini SV Andrade FB . Innate immune receptors in platelets and platelet-leukocyte interactions. J Leukoc Biol (2020) 108(4):1157–82. doi: 10.1002/JLB.4MR0620-701R 32779243

[46] Desai C Koupenova M Machlus KR Sen Gupta A . Beyond the thrombus: Platelet-inspired nanomedicine approaches in inflammation, immune response, and cancer. J Thromb Haemost (2022) 20(7):1523–34. doi: 10.1111/jth.15733 PMC9321119 35441793

[47] Kolaczkowska E Kubes P . Neutrophil recruitment and function in health and inflammation. Nat Rev Immunol (2013) 13(3):159–75. doi: 10.1038/nri3399 23435331

[48] Matsukane R Watanabe H Minami H Hata K Suetsugu K Tsuji T . Continuous monitoring of neutrophils to lymphocytes ratio for estimating the onset, severity, and subsequent prognosis of immune related adverse events. Sci Rep (2021) 11(1):1324. doi: 10.1038/s41598-020-79397-6 33446685 PMC7809015

[49] Jonsson AH Zhang F Dunlap G Gomez-Rivas E Watts GFM Faust HJ . Granzyme k(+) Cd8 T cells form a core population in inflamed human tissue. Sci Transl Med (2022) 14(649):eabo0686. doi: 10.1126/scitranslmed.abo0686 35704599 PMC9972878

