# Supplementary material for: Blood cell counts can predict adverse events of immune checkpoint inhibitors: A systematic review and meta-analysis
Source: Front Immunol. 2023 Mar 7;14:1117447. doi: 10.3389/fimmu.2023.1117447 (PMC10029759; doi:10.3389/fimmu.2023.1117447)
Supplement: Supplementary file 1 [file DataSheet_1.pdf]

Supplementary Material

Blood cell counts can predict adverse effects of immune checkpoint inhibitors: a systematic review and meta-analysis

1 Supplementary Table

1.1 Supplementary Table 1: Study characteristics

Title	Author	Year	Country	Journal	Patient number	Research type		Type of irAEs	Risk Factors
Association of baseline peripheral-blood eosinophil count with immune checkpoint inhibitor-related pneumonitis and clinical outcomes in patients with non-small cell lung cancer receiving immune checkpoint inhibitors	Xiangling Chu	2020	China	Lung Cancer	300	Retrospective analysis	single center	ICI-pneumonitis	Absolute eosinophil count(AEC)
Association between pretreatment neutrophil-to-lymphocyte ratio and immune-related adverse events due to immune checkpoint inhibitors in patients with non-small cell lung cancer	Airi Fujimoto	2021	Japan	Thoracic Cancer	115	Retrospective analysis	single center	Any irAEs	Neutrophil-to-lymphocyte ratio(NLR)
Absolute eosinophil count may be an optimal peripheral blood marker to identify the risk of immune-related adverse events in advanced malignant tumors treated with PD-1/PD-L1 inhibitors: a retrospective analysis	Yan Ma	2022	China	World journal of surgical oncology	95	Retrospective analysis	single center	Any irAEs	Absolute eosinophil count(AEC),neutrophil-to-lymphocyte ratio(NLR),platelet-to-lymphocyte ratio(PLR)
Eosinophil counts can be a predictive marker of immune checkpoint inhibitor-induced secondary adrenal insufficiency: a retrospective cohort study	Shinobu Takayasu	2022	Japan	Scientific Reports	525	Retrospective analysis	single center	Endocrine-related irAEs	Absolute eosinophil count(AEC),neutrophil-to-lymphocyte ratio(NLR),platelet-to-lymphocyte ratio(PLR)
Association Between Immune-related Adverse Events and Clinical Outcome Following Nivolumab Treatment in Patients With Metastatic Renal Cell Carcinoma	Kazuo Kobayashi	2020	Japan	In vivo	53	Retrospective analysis	single center	Any irAEs	Neutrophil-to-lymphocyte ratio(NLR), platelet:lymphocyte ratio(PLR)
Prediction of immune-related adverse events in non-small cell lung cancer patients treated with immune checkpoint inhibitors based on clinical and hematological markers: Real-world evidence	Huiru Xu	2022	China	Experimental cell research	667	Retrospective analysis	two centres	Any irAEs	Absolute basophils counts(ABC),absolute eosinophil count(AEC),serum albumin level,absolute lymphocyte count(ALC),alanine transaminase,alkaline phosphatase,absolute monocyte count(AMC);absolute neutrophil count(ANC)
Platelet-to-lymphocyte and neutrophil-to-lymphocyte ratios are associated with the efficacy of immunotherapy in stage III/IV non-small cell lung cancer	Xiaojuan Lu	2022	China	Oncology letters	133	Retrospective analysis	single center	Any irAEs	Platelet:lymphocyte ratio(PLR),neutrophil-to-lymphocyte ratio(NLR)
Correlations between peripheral blood biomarkers and clinical outcomes in advanced non-small cell lung cancer patients who received immunotherapy-based treatments	Yuequan Shi	2021	China	Translational Lung Cancer Research	103	Retrospective analysis	single center	Any irAEs	Absolute lymphocyte count(ALC),Neutrophil-to-lymphocyte ratio(NLR),platelet:lymphocyte ratio(PLR)
Neutrophil-to-Lymphocyte Ratio Predicts Development of Immune-Related Adverse Events and Outcomes from Immune Checkpoint Blockade: A Case-Control Study	Pei Yi Lee	2021	Singapore	Cancers(Basel)	147	Retrospective analysis	single center	Any irAEs	Neutrophil-to-lymphocyte ratio(NLR),platelet:lymphocyte ratio(PLR)
Association of Blood Biochemical Indexes and Antibiotic Exposure With Severe Immune-related Adverse Events in Patients With Advanced Cancers Receiving PD-1 Inhibitors	Lijun Zhao	2022	China	Journal of Immunotherapy	168	Retrospective analysis	single center	Any irAEs	Neutrophil-to-lymphocyte ratio(NLR)
Peripheral Blood Markers Identify Risk of Immune-Related Toxicity in Advanced Non-Small Cell Lung Cancer Treated with Immune-Checkpoint Inhibitors	Alberto Pavan	2019	Italy	Oncologist	184	Retrospective analysis	two centres	Any irAEs	Neutrophil-to-lymphocyte ratio(NLR),platelet:lymphocyte ratio(PLR)
Absolute Lymphocyte Count Predicts Immune-Related Adverse Events in Patients With Non-Small-Cell Lung Cancer Treated With Nivolumab Monotherapy: A Multicenter Retrospective Study	Saeka Egami	2021	Japan	Frontiers in Oncology	171	Retrospective analysis	Multicenter	Any irAEs	Absolute lymphocyte count (ALC), neutrophil-to-lymphocyte ratio (NLR), and lymphocyte-to-monocyte ratio (LMR)
Peripheral blood biomarkers predict immune-related adverse events in non-small cell lung cancer patients treated with pembrolizumab: a multicenter retrospective study	Saeka Egami	2021	Japan	Journal of Cancer	92	Retrospective analysis	Multicenter	Any irAEs	Absolute lymphocyte count (ALC), neutrophil-to-lymphocyte ratio (NLR), and lymphocyte-to-monocyte ratio (LMR),platelet:lymphocyte ratio(PLR)
Peripheral absolute eosinophil count identifies the risk of serious immune-related adverse events in non-small cell lung cancer	Yan Wu	2022	China	Frontiers in Oncology	213	Retrospective analysis	single center	Grades 3-5 AEs	Peripheral absolute eosinophil count (AEC)
Prognostic significance of the neutrophil-to-lymphocyte ratio and platelet-to-lymphocyte ratio for advanced non-small cell lung cancer patients with high PD-L1 tumor expression receiving pembrolizumab	Doran Ksienski	2021	Canada	Traditional Lung Cancer Research	220	Retrospective analysis	single center	Any irAEs	Neutrophil-to-lymphocyte ratio(NLR),platelet:lymphocyte ratio(PLR)
Correlation between blood cell count and outcome of melanoma patients treated with anti-PD-1 antibodies	Yoshiyuki Nakamura	2019	Japan	Japanese Journal of Clinical Oncology	45	Retrospective analysis	single center	Any irAEs	Absolute neutrophil count (ANC),absolute lymphocyte count (ALC),absolute monocyte count (AMC),neutrophil:lymphocyte ratio (NLR) ,peripheral absolute eosinophil count (AEC)
Peripheral Blood Biomarkers Predictive of Efficacy Outcome and Immune-Related Adverse Events in Advanced Gastrointestinal Cancers Treated with Checkpoint Inhibitors	Zhening Zhang	2022	China	Cancers(Basel)	243	Retrospective analysis	single center	Any irAEs	Platelet:lymphocyte ratio(PLR), neutrophil-to-lymphocyte ratio (NLR), and lymphocyte-to-monocyte ratio (LMR)
Analysis of characteristics and predictive factors of immune checkpoint inhibitor-related adverse events	Rilan Bai	2021	China	Cancer Biology & Medicine	105	Retrospective analysis	single center	Any irAEs	Relative lymphocyte count(RLC),platelet,absolute eosinophil count(AEC)

1.2 Supplementary Table 2: Subgroup analysis

	Subgroup analysis		Study number	OR	LCI	UCI	I2	P value
NLR with cutoff value	country							
		China	2	0.96	0.38	2.40	0.00%	0.484
		Japan	3	3.40	1.82	6.32	0.00%	0.573
	patient number							
		100-200	2	2.46	1.42	4.25	0.00%	0.308
		< 100	3	3.13	1.29	7.60	52.10%	0.124
	cutoff value	2.5-3.5	4	2.13	1.34	3.40	0.05	0.569
NLR without cutoff value	country							
		China	3	0.98	0.91	1.05	60.20%	0.081
	patient number							
		100-200	3	0.88	0.75	1.04	76.40%	0.014
PLR with cutoff value	country							
		China	2	0.86	0.44	1.67	37.60%	0.206
		Japan	2	3.78	1.84	7.78	0.00%	0.379
	patient number							
		< 100	3	1.92	1.07	3.43	81.10%	0.005
PLR without cutoff value	country							
		China	3	1	1	1	58.20%	0.091
	patient number							
		100-200	3	1	0.99	1	59.70%	0.084

2 Supplementary Figure

2.1 Supplementary Figure 1: Quality assessment of the studies included in the meta-analysis

	Study Participation	Study Attrition	Prognostic Factor Measurement	Outcome Measurement	Study Confounding	Statistical Analysis and Reporting	Overall Evaluation
Chu2020	H	M	M	L	M	M	H
Fujimoto2021	L	M	M	M	M	L	M
Ma2022	L	M	L	L	M	L	L
Takayasu2022	H	M	H	L	M	M	H
Kobayashi2020	L	M	M	L	M	L	M
Xu2022	M	M	L	M	L	L	L
Lu2022	H	M	M	H	L	L	H
Shi2021	M	M	L	L	L	L	L
Lee2021	M	M	M	M	H	L	H
Bai2021	L	M	M	L	L	L	L
Pavan2019	L	M	M	L	H	L	M
Egami2021(19)	L	M	L	L	M	L	L
Egami2021(20)	L	M	M	L	H	L	M
Wu2022	M	M	H	M	H	L	H
Ksienski2021	L	M	M	M	H	L	H
Nakamura2019	M	M	M	L	H	L	H
Zhang2022	M	M	L	L	M	L	M
Zhao2022	M	M	L	L	M	L	M

Green: L (low risk of bias)

Yellow: M (moderate risk of bias),

Red: H (high risk of bias)
