# Supplementary material for: Blood cell counts can predict adverse events of immune checkpoint inhibitors: A systematic review and meta-analysis
Source: Front Immunol. 2023 Mar 7;14:1117447. doi: 10.3389/fimmu.2023.1117447 (PMC10029759; doi:10.3389/fimmu.2023.1117447)
Supplement: Supplementary file 2 [file DataSheet_2.pdf]

Search strategy

Pubmed	(((("Immune Checkpoint Inhibitors"[Mesh]) OR (immune checkpoint) OR(immunetherapy)OR (Ipilimumab) OR (nivolumab) OR (pembrolizumab)OR (PD-L1)OR (Programmed Death-Ligand 1)OR (CTLA-4)OR (Cytotoxic T-Lymphocyte-Associated Protein 4)OR (PD-1)OR (Programmed Cell Death Protein 1)OR (durvalumab) OR (atezolizumab) OR (avelumab) OR (Toripalimab) OR (Sintilimab) OR (Camrelizumab) OR (Tislelizumab) OR (Serplulimab) OR (Penpulimab) OR (Cejemly) OR (Envafolimab)) AND ((Immune checkpoint inhibitor-related adverse reaction*) OR (irAE*)OR(immune-related toxicity)OR (drug-related adverse reaction*))) AND (("Risk Factors"[Mesh]) OR (risk factor*)OR (predicto*)OR (exposur*)OR(risk))) AND ((blood) OR (blood biomaker*) OR (blood marker)OR (eosinophil) OR (neutrophil) OR (lymphocyte) OR (monocyte) OR (Platelet) OR (AEC) OR (NLR) OR (PLR) OR (ANC) OR (ALC) OR (AMC) OR (PLT))
Wed of Science	TS=("durvalumab" OR "atezolizumab" OR "avelumab" OR "Toripalimab" OR "Sintilimab" OR "Camrelizumab" OR "Tislelizumab" OR "Serplulimab" OR "Penpulimab" OR "Cejemly" OR "Envafolimab" OR "Immune Checkpoint Inhibitors"OR "Ipilimumab"OR "nivolumab"OR "pembrolizumab"OR "PD-L1"OR "Programmed Death-Ligand 1"OR "CTLA-4"OR "Cytotoxic T-Lymphocyte-Associated Protein 4"OR "PD-1"OR "Programmed Cell Death Protein 1"OR"immune checkpoint"OR"immunetherapy")) AND TS=("Risk Factors"OR "risk factor*"OR "predicto*"OR "exposur*"OR"risk")) AND TS=("Immune checkpoint inhibitor-related adverse reaction*"OR "irAE*"OR "drug-related adverse reaction*"OR"(immune-related toxicity")) AND TS=("blood" OR "blood biomaker*" OR"eosinophil" OR "neutrophil" OR "lymphocyte" OR "monocyte" OR "Platelet"OR"blood marker"OR"AEC"OR"NLR"OR"PLR"OR"ANC"OR"ALC"OR"AMC"OR"PLT")
Embase	(durvalumab):ab,ti OR ((atezolizumab):ab,ti) OR ((avelumab):ab,ti) OR ((Toripalimab):ab,ti) OR ((Sintilimab):ab,ti) OR ((Camrelizumab):ab,ti) OR ((Tislelizumab):ab,ti) OR ((Serplulimab):ab,ti) OR ((Penpulimab):ab,ti) OR ((Cejemly):ab,ti) OR ((Envafolimab):ab,ti) OR (('Immune Checkpoint Inhibitors')/exp) OR ((Ipilimumab):ab,ti) OR ((nivolumab):ab,ti) OR ((pembrolizumab):ab,ti) OR ((PD-L1):ab,ti) OR (('Programmed Death-Ligand 1'):ab,ti) OR ((CTLA-4):ab,ti) OR (('Cytotoxic T-Lymphocyte-Associated Protein 4'):ab,ti) OR ((PD-1):ab,ti) OR (('Programmed Cell Death Protein 1'):ab,ti) OR (('immune checkpoint'):ab,ti) OR ((immunetherapy):ab,ti) AND('risk factor')/exp OR (('risk factors'):ab,ti) OR ((predicto*):ab,ti) OR ((exposur*):ab,ti) OR ((risk):ab,ti)AND('Immune checkpoint inhibitor-related adverse reaction*'):ab,ti OR ((irAE*):ab,ti) OR (('drug-related adverse reaction*'):ab,ti) OR (('immune-related toxicity'):ab,ti)AND('blood biomaker'):ab,ti OR ((blood):ab,ti) OR ((eosinophil):ab,ti) OR ((neutrophil):ab,ti) OR ((lymphocyte):ab,ti) OR ((monocyte):ab,ti) OR ((Platelet):ab,ti) OR (('blood marker'):ab,ti) OR ((AEC):ab,ti) OR ((NLR):ab,ti) OR ((PLR):ab,ti) OR ((ANC):ab,ti) OR ((ALC):ab,ti) OR ((AMC):ab,ti) OR ((PLT):ab,ti)
